# A standard set of outcome measures for the comprehensive assessment of osteogenesis imperfecta

**DOI:** 10.1186/s13023-021-01682-y

**Published:** 2021-03-20

**Authors:** Wouter Nijhuis, Anton Franken, Kara Ayers, Chantal Damas, Lars Folkestad, Antonella Forlino, Paolo Fraschini, Claire Hill, Guus Janus, Richard Kruse, Lena Lande Wekre, Lieve Michiels, Kathleen Montpetit, Leonardo Panzeri, Valerie Porquet-Bordes, Frank Rauch, Ralph Sakkers, Jean-Pierre Salles, Oliver Semler, Jony Sun, Michael To, Laura Tosi, Yangyang Yao, Eric Hiu Kwong Yeung, Lidiia Zhytnik, Maria Carola Zillikens, Marjolein Verhoef

**Affiliations:** 1 University Medical Center Utrecht, Utrecht, The Netherlands; 2 Isala Zwolle, Utrecht, The Netherlands; 3 Cincinnati Children’s Hospital Medical Center, Cincinnati, USA; 4 Shriners Hospitals for Children, Montreal, Canada; 5 University of Southern Denmark, Odense, Denmark; 6 University of Pavia, Pavia, Italy; 7 Rizzoli Institute Bologna, Milano, Italy; 8 Sheffields Children’s NHS Trust Foundation, Sheffield, UK; 9 Nemours/Alfred Dupont Hospital for Children, Delaware, USA; 10 TRS National Resource Center for Rare Disorders, Sunnaas Rehabilitation Hospital, Oslo, Norway; 11 ZOI (Flemish OI Association), Brussels, Belgium; 12 Advisor Care4BrittleBones Foundation, Montreal, Canada; 13 AS.IT.O.I. (Italian OI Association), Rome, Italy; 14 CHU de Toulouse, Toulouse, France; 15 Department of Paediatrics, University of Cologne, Cologne, Germany; 16 China-Dolls Center for Rare Disorders (CCRD), Bejing, China; 17 The University of Hong Kong - Shenzhen Hospital, Hong Kong SAR, Shenzhen, China; 18 Children’s National Hospital, Washington, USA; 19 Shandong Provincial Hospital, Jihan, China; 20 Tartu University Hospital, Tartu, Estonia; 21 University Medical Center Erasmus Rotterdam, Rotterdam, The Netherlands

**Keywords:** Osteogenesis imperfecta, Brittle bone disease, Continuous quality improvement, Learning health care, Outcomes, Patient-reported outcomes measures, Value-based health care, Clinical outcome measures

## Abstract

**Background:**

Osteogenesis Imperfecta (OI) is a genetic disorder also known as ‘brittle bone disease’. The clinical manifestation of OI shows a wide variation. Therefore, care for patients with OI requires an interdisciplinary approach. The effectiveness of particular interventions and treatment protocols of interdisciplinary teams is not clear due to a non-standardized and wide variation of patient outcomes thus making the comparison of outcome measures available in the literature difficult. It is only by agreeing on a common, standard set of outcome measures for the comprehensive appraisal of OI that comparisons across interdisciplinary treatment centers for OI will be possible in the future.

**Methods:**

The Key4OI international interdisciplinary working group of 27 members used a consensus-driven modified Delphi approach to develop a set of global outcome measures for patients with OI. The International Classification of Functioning, Disability and Health (ICF), was used to define domains and organize the outcomes from the literature search. After reviewing the outcomes extracted from the literature, trials and registries, the working group agreed on a final selection of domains and their definition (ICF definition as well as a lay description). These domains were then presented to the focus groups who prioritized the outcome domains by taking into account the items important to the OI community. All content was collected and analyzed and final domains were determined. A consensus of appropriate measuring instruments for each domain was reached with Delphi rounds. The entire approach was in line with the International Consortium for Health Outcomes Measurement ICHOM methodology.

**Results:**

More than 400 different outcome measures were identified in our literature search. After three Delphi rounds, 24 domains were selected. After the focus group sessions, the number of domains were reduced to 15. A consensus was reached on the measuring instruments to cover these domains for both children and adults.

**Conclusion:**

The Key4OI project resulted in standard set of outcome measures focused on the needs and wishes of individuals with OI and their families. This outcome set will enable healthcare teams and systems to compare and to improve their care pathways and quality of care worldwide. Further studies are needed to evaluate the implementation of this standardized outcome set.

## Background

In evidence-based health care, a key determining factor for research and evaluation of clinical care is the choice of outcomes. Outcome measuring instruments must be reliable, valid, and feasible [1,2]. Trials using inappropriate instruments may overestimate, underestimate, or overlook the effect of an intervention [3]. Standardization is necessary in order to allow cross-trial comparison in systematic reviews. Similarly, meta-analyses, needed for evidence-based clinical practice guidelines, are only possible with validated and comparable outcomes [4]. Osteogenesis Imperfecta (OI) is a genetic disorder also known as ‘brittle bone disease’ Autosomal dominant mutations in the type I collagen coding genes (COL1A1 and COL1A2) affect the collagen structure in the majority of OI patients. More recently recessive, dominant and X-linked defects in a wide variety of genes encoding proteins involved in type I collagen synthesis have been shown to cause osteogenesis imperfecta [5]. The current non-standardized and wide variation of outcomes in studies on patients with OI makes comparison of data difficult. Many studies and registries on OI exhibit marked heterogeneity in terms of what domains are measured and how the domains are defined. Outcome research in OI is especially difficult because of the inherent complexity of the condition. OI does not only affect bone but all tissues containing collagen type I as well. The clinical manifestations vary widely between the different types of OI ranging from patients who have mild symptoms with a normal life expectancy to intrauterine death [6–8]. Even within the same type of OI there is a wide spectrum of clinical manifestations.

From birth to young adulthood, a child grows and develops in all domains such as mobility, self-care and participation. In addition, there is development toward independence and maturity. For all these stages, with their own particular focus and perspective, outcomes that are comparable worldwide, are important for further improvement of high-quality interdisciplinary care [9].

The challenge will be to define a set of outcomes for patients with OI that covers all the important domains, especially since the relevant outcome data will be different for different ages. To meet this challenge, the Care4BrittleBones foundation initiated Key4OI, a project to develop a minimum standard set of outcomes and associated measures for the comprehensive appraisal of OI that would reflect the complexity of interdisciplinary OI care and focus on what matters most to patients with OI and their families.

## Objectives

The primary objective of this initiative was to reach an international, interdisciplinary consensus for a standard set of outcomes and associated measuring instruments for the care of individuals with OI, based on what is important to both experts and patients. This standard set would be comprehensive enough to cover the full range of OI care, yet practical enough for sustainable implementation. This will permit teams around the world to measure their performance in a consistent way. This will support longitudinal and cross-sectional comparison of outcomes between centers that serve OI—populations in different environments.

## Methods

A modified Delphi technique was used to develop a minimal standard outcome set. The Delphi technique is an iterative multi stage process to actively transform opinion into group consensus [10, 11].

This consensus must be based on data derived from all stakeholders involved in the care of individuals with OI including the people with OI themselves. In order to achieve this, an assembly of three groups from the OI community was formed, consisting of a lead team, an expert team and focus groups. In each country an ethical review was conducted and ethical approval was obtained where required.

The lead team consisted of six professionals, five were members of a pediatric or adult OI interdisciplinary team and the sixth was the coordinator from the non-government organization (NGO) Care4BrittleBones. The role of the lead team was to drive the overall project, spearhead the initial research and literature search, and prepare all materials for the video  conferences, expert team meetings and focus groups. The expert team consisted of 21 professionals. Membership included internationally recognized professionals, as well as representatives of patient organizations from different countries. Overall, eight countries on three continents were represented. Clinical disciplines represented included orthopedic surgery, rehabilitation, genetics, pediatrics, psychology, physiotherapy, occupational therapy and endocrinology. The background of the professionals who participated is shown in Table 1. The role of the expert team was to advise and provide input on materials presented by the lead team, engage with others and work towards consensus by participating in Delphi rounds.

**Table 1:** Details of expert team members

Role	Name	Surname	Title	Location	Prof. background	Additional perspective	Emailadress	Institute
Lead Team	Anton FRANKEN	Franken	MD, PhD	Zwolle, the Netherlands	Endocrinologist		a.a.m.franken@isala.nl	Isala Hospital
Lead Team	Guus JANUS	Janus	MD, PhD	Zwolle, the Netherlands	Orthopaedic Surgeon		a.j.m.janus@isala.nl	Isala Hospital
Lead Team	Wouter NIJHUIS	Nijhuis	MD	Utrecht, the Netherlands	Orthopaedic Surgeon		W.H.Nijhuis-2@umcutrecht.nl	University Medical Center Utrecht
Lead Team	Ralph SAKKERS	Sakkers	MD, PhD	Utrecht, the Netherlands	Orthopaedic Surgeon	OIFE Medical Advisory Board Member	R.Sakkers@umcutrecht.nl	University Medical Center Utrecht
Lead Team	Marjolein VERHOEF	Verhoef	MD, PhD	Utrecht, the Netherlands	Rehabilitation Physician		M.Verhoef-19@umcutrecht.nl	University Medical Center Utrecht
Lead Team (Project Mgt)	Dagmar MEKKING	Mekking	PhD	The Hague, the Netherlands	Project Manager	Director Care4BrittleBones Foundation, OI-community	dagmar.mekking@care4brittlebones.org	Care4BrittleBones Foundation
Expert Team	Kara AYERS	Ayers	PhD	Cincinnati, USA	Psychologist	Vice-President OIF Board of Directors, OI-community	Kara.Ayers@cchmc.org	Cincinnati Children's Hospital Medical Center
Expert Team	Chantal DAMAS	Damas	BSc	Montreal, Canada	Physiotherapist	Coordinator Center of Excellence	CDamas@shrinenet.org	Shriners Hospitals for Children, Montreal
Expert Team	Lars FOLKESTAD	Folkestad	MD, PhD	Odense, Denmark	Endocrinologist	OIFE Medical Advisory Board Member, Care4BrittleBones Foundation Advisory Board Member	lfolkestad@health.sdu.dk	University of Southern Denmark
Expert Team	Antonella FORLINO	Forlino	PhD	Pavia, Italy	Biochemistry, Genetics	OIFE Medical Advisory Board Member, Care4BrittleBones Foundation Advisory Board Member	antonella.forlino@unipv.it	University of Pavia
Expert Team	Paolo FRASCHINI	Fraschini	MD	Milano, Italy	Rehabilitation Physician		paolofraschini@gmail.com	Rizzoli Institute Bologna Vele affliaties
Expert Team	Claire HILL	Hill	PhD, MCSP PGCE	Sheffield, UK	Physiotherapist	OIFE Medical Advisory Board Member	clairehill3216@gmail.com	Sheffields Children's NHS trust foundation
Expert Team	Richard KRUSE	Kruse	DO, MBA	Delaware, USA	Orthopaedic Surgeon		Richard.Kruse@nemours.org	Nemours/Alfred Dupont Hospital for children
Expert Team	Lena LANDE WEKRE	Wekre	MD, PhD	Oslo, Norway	Rehabilitation Physician	OIFE Medical Advisory Board Member, Care4BrittleBones Foundation Advisory Board Member	LENALW@sunnaas.no	TRS National Resource Center for Rare Disorders, Sunnaas Rehabilitation Hospital
Expert Team	Lieve MICHIELS	Michiels	PhD	Belgium	not applicable	Board member ZOI, Belgium OI Association, OI Community	dillen.michiels@skynet.be	ZOI (Flemish OI Association)
Expert Team	Kathleen MONTPETIT	Montpetit	MScOT	Montreal, Canada	Occupational Therapist, retired	Advisor Care4BrittleBones Foundation	kathleenmontpetit@yahoo.ca	Care4BrittleBones Foundation
Expert Team	Leonardo PANZERI	Panzeri	not applicable	Italy	not applicable	President Italian OI Association AS.IT.O.I., OI Community	leonardo.panzeri@asitoi.it	AS.IT.O.I. (Italian OI Association)
Expert Team	Valerie PORQUET-BORDES	Bordes	MD, PhD	Toulouse, France	Pediatrician	Care4BrittleBones Foundation Advisory Board Member	porquet-bordes.v@chu-toulouse.fr	CHU de Toulouse
Expert Team	Frank RAUCH	Rauch	MD, PhD	Montreal, Canada	Pediatrician		frank.rauch@mcgill.ca	Shriners Hospitals for Children, Montreal
Expert Team	Jean-Pierre SALLES	Salles	MD, PhD	Toulouse, France	Physiopathologist		salles.jp@chu-toulouse.fr	CHU de Toulouse
Expert Team	Oliver SEMLER	Semler	MD	Cologne, Germany	Pediatrician	OIFE Medical Advisory Board Member, Care4BrittleBones Foundation Advisory Board Member, OI-community	joerg.semler@uk-koeln.de	University of Cologne, Department of Paediatrics
Expert Team	Jony SUN	Sun	MSW, Project Manager	Beijing, China	Social Worker	China-Dolls Center for Rare Disorders (CCRD), OI Community	82,456,214@qq.com	China-Dolls Center for Rare Disorders(CCRD)
Expert Team	Michael TO	To	MD, PhD	Hong Kong SAR and Shenzhen, China	Orthopaedic Surgeon		mikektto@hku.hk	The University of Hong Kong—Shenzhen Hospital
Expert Team	Laura TOSI	Tosi	MD	Washington, USA	Orthopaedic Surgeon	OIF Medical Adviory Board	LTOSI@childrensnational.org	Children’s National Hospital, Washington, DC
Expert Team	Yangyang YAO	Yao	MD	Shandong Provincial Hospital, China	Orthopaedic Surgeon		15,165,313,344@126.com	Shandong Provincial Hospital
Expert Team	Eric Hiu Kwong YEUNG	Yeung	MSc, BSc	Hong Kong SAR and Shenzhen, China	Physiotherapist		ericyhk@hku.hk	The University of Hong Kong—Shenzhen Hospital
Expert Team	Lidiia ZHYTNIK	Zhytnik	PhD	Tartu, Estonia	Genetics, Biomedicine	OIFE Medical Advisory Board Member, Care4BrittleBones Foundation Advisory Board Member, OI-community	Lidiia.zhytnik@ut.ee	Tartu University Hospital
Expert Team	Maria Carola ZILLIKENS	Zillikens	Prof. MD, PhD,	Rotterdam, the Netherlands	Endocrinologist		m.c.zillikens@erasmusmc.nl	University Medical Center Erasmus Rotterdam

Focus groups of either adults or children (ages 10–18 years) were held in 11 different countries over 3 continents to determine which domains matter most to the OI community worldwide and were set up with the help of local OI patient organizations. The people with OI described the level of severity of their condition (mild, moderate or severe) as well as their ambulatory status. No medical confirmation was asked. We did not record the type of OI. All focus groups consisted of a range of severity and when possible, each country held an adult as well as a child focus group. The minimum number of participants in a focus group was five for children and eight for adults. Only individuals with OI themselves were included and not their parents. Literature on focus groups advises clustering children and youth per age group, allowing a discussion among peers. Teens show an increased ability in abstract reasoning, problem solving and decision making [12].Thus focus groups were with children from 10 to 18 years old. In addition we asked the adults to reflect on their youth in order to gain further information about the younger age groups.

In order to reach consensus on every decision, modified Delphi rounds were held with the expert team. The lead team had no vote in the Delphi rounds. Over a period of one and a half years, the lead team together with the expert team held a total of 20 videoconferences. Also a final face to face meeting took place during an international conference in November 2019. Each meeting had at least 80% participation.

### Process

The lead team conducted a literature search to identify all outcome domains reported in the medical literature. Broad search terms were used in order not to overlook any domains. The search terms were Brittle Bone Disease and Osteogenesis Imperfecta. Inclusion criteria were original research articles, publications issued in the past five years, registries, multicenter studies, clinical trials and publications in peer-reviewed journals. Exclusion criteria were articles not available in English, the inability to obtain the full-text article, abstracts, editorials, commentaries, letters, and case reports. All outcomes reported in the included articles together with the outcomes collected in three ongoing unpublished trials (TOPAZ, BOOSTB4 and Mereo) and four known registries (UMC Utrecht, Isala, USA linked clinical research and Cologne) were collected into one database.

**Fig. 1:**
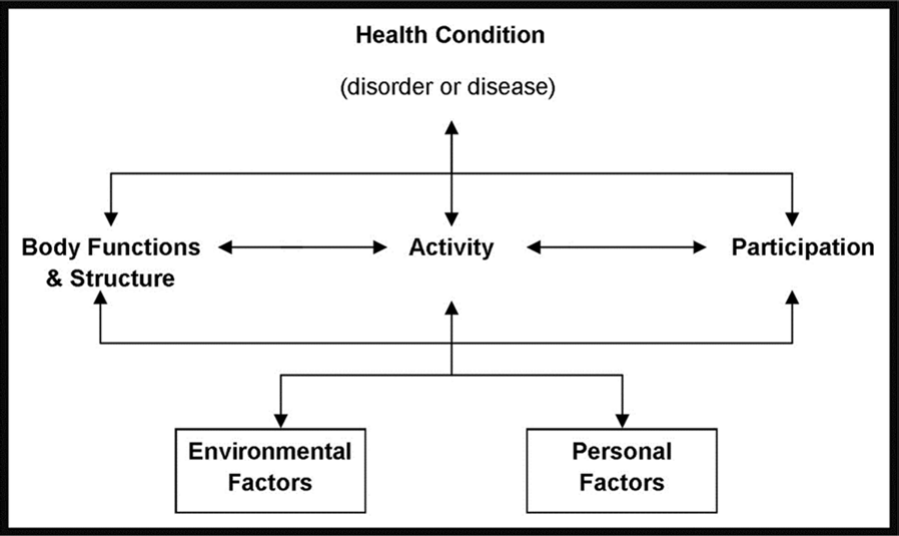
Model of the international classification of functioning, disability and health (ICF). The ICF conceptualizes a person's level of functioning as a dynamic interaction between her or his health conditions, environmental factors, and personal factors [13]

Next step was the aggregation of the data following the structure of the International Classification of Functioning, Disability and Health (ICF). The ICF is an international classification that describes a health condition in terms of body functions and structure, activities and participation and personal and environmental factors as well as how they are interrelated [13]. All 191 WHO Member States officially endorsed the ICF in 2001 as the international standard to describe and measure health and disability (Fig. 1).

After reviewing the outcomes extracted from the literature, trials and registries, the lead team identified domains and the expert team proceeded to prioritize these domains. After three modified Delphi rounds, the expert team agreed on a final selection of these domains and their definition (ICF definition as well as a lay description). These domains were then presented to the focus groups after translation into the languages appropriate for the countries in which the focus groups were held.

The process for the focus groups was described in a detailed protocol including a standard set of slides and a scoring sheet to identify and prioritize the outcome domains taking into account the items of importance for the OI community and their wishes and hopes for the future per domain. Domains were ranked and then added or removed as per group consensus. The standardized approach was discussed at the outset with the national OI patient organizations in each country in order to respect the cultural aspects and to ensure that in each cultural setting the participants would feel comfortable to speak up in order to obtain outcomes of consistent quality. All content was collected and analyzed by the lead team and the final domains were determined by the expert team. Ethical review for the focus groups was obtained according to local requirements.

The subsequent step was the selection of the appropriate measuring instruments for each domain by the lead team, using a database and library of measuring instruments based on the literature and clinical practice. Guided by the feedback from the focus groups, sustainability and validity of the measures per domain, a pre-selection was made. Practical issues such as time required, resources needed and availability of the instrument were taken into account. A generic measuring instrument was preferred over a disease specific instrument to enable generic disease comparison in the future. A single instrument covering multiple domains with different subscales was preferred over using multiple instruments. This pre-selection was evaluated by the expert team who added additional measuring instruments and personal feedback on the selection and use of the measuring instruments from their own clinical practice. In a next Delphi round, experts were asked to rate the instruments on a 9-point scale. A minimum percentage of 80% with a score 7, 8 or 9 was required for the final confirmation of a measuring instrument. A score of 1, 2 and 3 in 80% of the responses lead to a final rejection of the measurement instrument. Mid-range scores were considered “non-conclusive”, discussed in the next expert meeting, and tabled in the next Delphi round. A participation rate of 80% of experts was required in the Delphi rounds. The entire approach was in line with the International Consortium for Health Outcomes Measurement ICHOM methodology [14].

## Results

### Selection of outcome domains

**Fig. 2:**
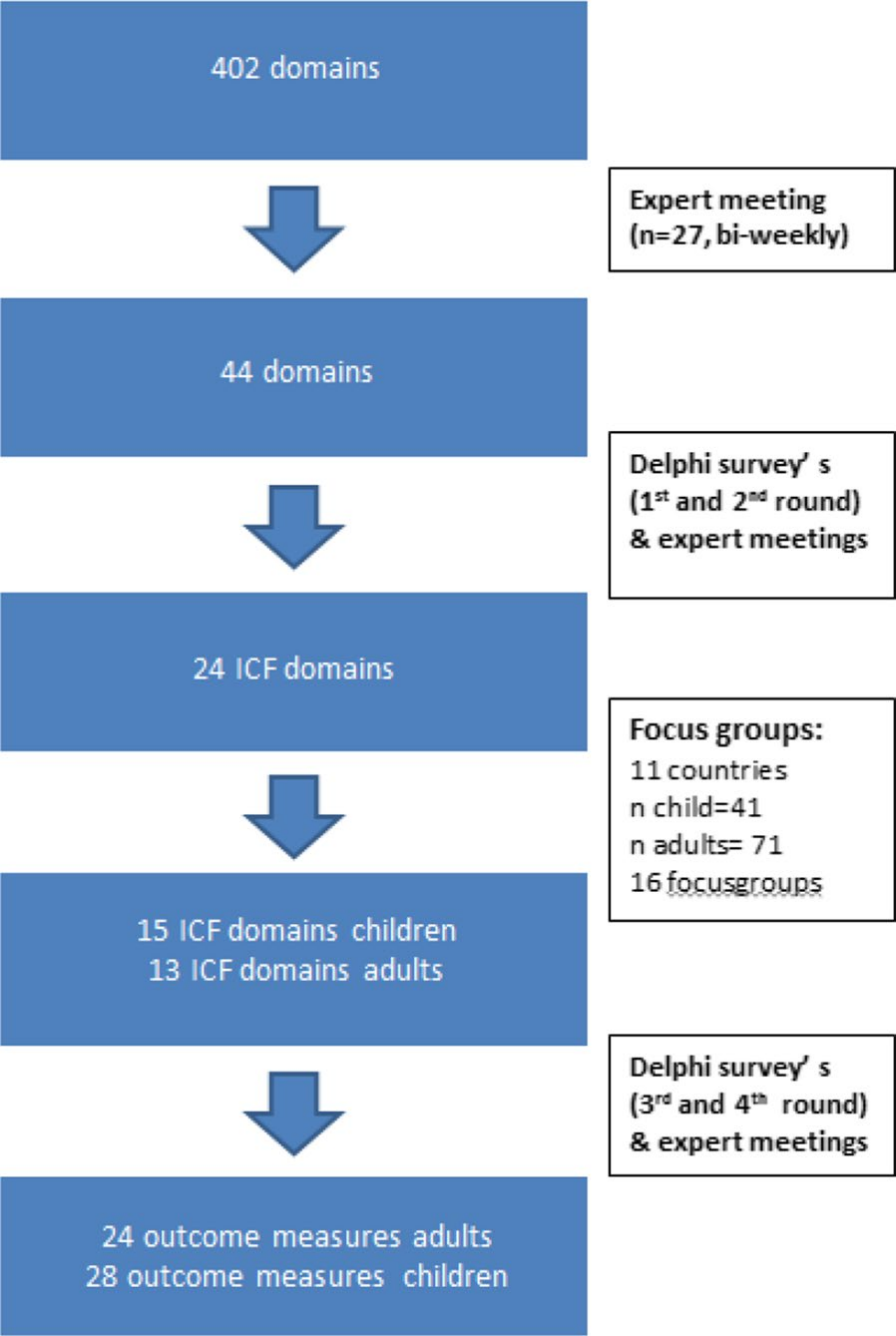
Flowchart outcome domains and measurements

The literature search yielded over 6000 hits including 19 trials, 16 multicenter studies, and 2 registry studies. After correction for duplicates, 49 articles were reviewed and resulted in a database of 402 different outcome domains. After reducing the 402 domains reported in the literature to 44, these domains were then prioritized by the expert team through 3 modified Delphi rounds and 24 domains were selected (Table 2). These 24 domains with the ICF definition as well as a lay description were then presented to the focus groups. An overview of the process of selecting the outcome domains and measurements can be found in the flowchart in Fig. 2.

**Table 2:**
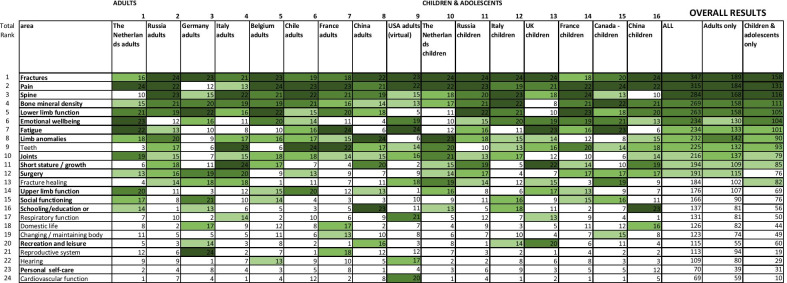
Selected domains

The results of ranking by all the focus groups. The  darker the background the higher the rating  of the domain. The bolded domains are the final selected domains based on the priorities indicated by the focus groups as well as the overal evaluation of the expert team. The domains recreation and leisure were combined in to one domain called participation

The focus groups for children had 41 participants with a mean age of 14, 1 (STDEV 2.32) (10–18 years of age) of whom 66% was female. Mobility level of the participants was 46% ambulant, 49% wheelchair and 5% both wheelchair and ambulant. The adult focus groups had 71 participants with a mean age of 33.7 (STDEV 12.3) 16–70 years of age. 63% of the adults were female. Mobility level was 28% ambulant, 49% wheelchair and 23% both wheelchair and ambulant. All focus groups had a mixture of different OI types. Focus groups were held in Belgium, Canada, Chile, China/Hong Kong, France, Germany, Italy, the Netherlands, Russia, UK and  USA. The cumulative time of discussion in these groups was 80 h.

The focus group resulted in a prioritized list of domains. In addition, 23 new issues appeared. For example, in the domain ‘pain’ often "the inability to work" was suggested. After discussion by the expert group the majority of the issues appeared to be covered by the domains initially selected and all issues were felt to be part of one of the 24 designated domains or part of the demographic profile (Table 3). Based on the priorities indicated by the focus groups as well as the overall evaluation of the expert team, the final number of domains was reduced to 15 for children and 13 for adults with OI (Table 2). All domains were structured according to the WHO ICF [13]  and categorized within 4 major themes; major events, clinical status, functioning and quality of life (Table 3).

**Table 3:** Demographic profile and outcome themes, four major outcome themes, domains and subdomains with the selected outcome measures

Patient population	Measure	Supporting information	Timing	Data source
*Patient demographic factors*			Diagnosis	Clinical
All patients	Date of birth	N/A		
	Gender	Gender at birth		
	Patient’s status	Patient alive or dead		
	If previous question is equal to “death”: Date of death	Patient’s date of death		
	First contact with specialized center	Date of the first contact with the specialized center		
	Age at onset	Age at which the symptoms/sign first appeared		
	Age at diagnosis	Age at which diagnosis was made		
*Family demographic factors*				
All patients	Sibling with OI	N/A		
	Father with OI	N/A		
	Mother with OI	N/A		
*Baseline clinical status*				
All patients	Diagnosis OI	ORPHA code		
	Diagnosis OI	Orphanet code		
	Genetics	Exact mutation International classification of HGVS (http://www.hgvs.org)/HGNC/OMIM code		
	Clinical OI type	Sillence classification (Type I–V)		

Patient population	Measure	Measurement tool	Timing (min)	Data source
*Major events*				
All patients	Fractures	The approximate number of historical fractures	Baseline	Self/parent reported
All patients		Patient reported fracture incidence	Children: at diagnosis and 3, 6, 8, 11, 15 years end of paediatric care (< 18 years) Adults: 18 years and every 5 years	Self/parent reported
All patients		Clinically evaluated fracture incidence		Clinical^a^
All patients		X-ray/radiologically confirmed fracture incidence		
Children		Mechanism (high energy trauma, low energy trauma, unknown)		
All patients		Treatment (surgical, conservative, no treatment)		
All patients		Fracture healing: duration < 2 months, 2–6 months, > 6 months, non-union		
Children	Surgery	Frequency per year		
All patients		Type of surgery		
All patients		Reason: Fracture/deformity		
All patients		Fracture healing: < 2 months, 2–6 months, > 6 months, non union		
All patients		Reoperation rate		

Patient population	Measure	Measurement tool	Timing (min)	Data source
Clinical status				
All patients	Bone mineral density	DEXA corrected for height	Children: yearly	Clinical
			Adults: 18 years and every 5 years	
		Medical treatment	Children: yearly	
			Adults: 18 years and every 5 years	
Children	Spine deformity	X-Ray Total Spine AP and Lateral, Cobb angles	Children: at diagnosis and 3, 6, 8, 11, 15 years end of paediatric care (< 18 years)	
Adults			Adults: baseline	
Children	Joints	Beighton (5 +)	Children: at diagnosis and 6, 8, 11, 15 years end of paediatric care (< 18 years)	
Adults			Adults: baseline	
Children	Limb anomalies	Leg length discrepancy: clinical	Children: at diagnosis and 3, 6, 8, 11, 15 years end of paediatric care (< 18 years)	
Children		Leg length discrepancy: AP and/or lateral Standing Long Leg X-rays	Children: baseline at 8 years	
Adults		Leg length discrepancy: clinical	Adults: baseline	
Children	Growth and weight	Length (overall height)	Children: yearly	Clinical^a^
Adults			Adults: baseline	
Children		Arm span	Children: yearly	
Adults			Adults: baseline	
Children		Weight	Children: yearly	
Adults			Adults: 18 years and every 5 years	
*Function*				
Children	Lower Limb	FMS	Children: at diagnosis and 3, 6, 8, 11, 15 years end of paediatric care (< 18 years)	Clinical
Children		PROMIS Pediatric Item Bank v2.0—Mobility—Short Form 8a & PROMIS Parent Proxy Item Bank v2.0—Mobility—Short Form 8a	Children: at diagnosis and 6, 8, 11, 15 years end of paediatric care (< 18 years)	Self reported: from 8 years and older Parent reported: all ages
All patients		30 s walk test	Children: at diagnosis and 6, 8, 11, 15 years end of paediatric care (< 18 years) Adults: 18 years and every 5 years	Clinical
Adults		PROMIS Item Bank v2.0—Physical Function—Short Form 8b	Adults: 18 years and every 5 years	Self reported
Children	Upper limb function	PROMIS Pediatric Item Bank v2.0—Upper Extremity—Short Form 8a & PROMIS Parent Proxy Item Bank v2.0—Upper Extremity—Short Form 8a	Children: at diagnosis and 6, 8, 11, 15 years end of paediatric care (< 18 years)	Self reported: from 8 years and older Parent reported: all ages
Adults		PROMIS Item Bank v2.0—Upper Extremity—Short Form 7a	Adults: 18 years and every 5 years	Self reported
Children	Selfcare	PROMIS Pediatric Item Bank v2.0—Upper Extremity—Short Form 8a & PROMIS Parent Proxy Item Bank v2.0—Upper Extremity—Short Form 8a	Children: at diagnosis and 6, 8, 11, 15 years end of paediatric care (< 18 years)	Self reported: from 8 years and older Parent reported: all ages
Adults		SUNNAAS ADL Index in adults	Adults: 18 years and every 5 years	Clinical
*Quality of life*				
Children	Pain	PROMIS Pediatric Item Bank v2.0—Pain Interference—Short Form 8a & PROMIS Parent Proxy Item Bank v2.0—Pain Interference—Short Form 8a	Children: at diagnosis and 6, 8, 11, 15 years end of paediatric care (< 18 years)	Self reported: from 8 years and older Parent reported: all ages
Adults		PROMIS Item Bank v1.0—Pain Interference—Short Form 8a	Adults: 18 years and then every 5 years	Self reported
Children		Faces Pain Rating Scale colored analogue scale	Children 6, 8, 11 years	Self reported
Adults		PROMIS Item Bank v.1.0—Pain Intensity—Scale	Adults: 18 years and then every 5 years	Self reported
Children	Emotional wellbeing	PROMIS Pediatric Item Bank v2.0—Anxiety—Short Form 8a & PROMIS Parent Proxy Item Bank v2.0—Anxiety—Short Form 8a	Children: at diagnosis and 6, 8, 11, 15 years end of paediatric care (< 18 years)	Self reported: from 8 years and older Parent reported: all ages
Children		PROMIS Pediatric Item Bank v2.0—Depressive Symptoms—Short Form 8a & PROMIS Parent Proxy Item Bank v2.0—Depressive Symptoms—Short Form 6a	Children: at diagnosis and 6, 8, 11, 15 years end of paediatric care (< 18 years)	Self reported: from 8 years and older Parent reported: all ages
Adults		PROMIS Item Bank v1.0—Emotional Distress—Anxiety—Short Form 8a	Adults: 18 years and then every 5 years	Self reported
Adults		PROMIS Item Bank v1.0—Emotional Distress—Depression–Short Form 8a	Adults: 18 years and then every 5 years	Self reported
Children	Fatigue	PROMIS Pediatric Item Bank v2.0—Fatigue—Short Form 10a & PROMIS Parent Proxy Item Bank v2.0—Fatigue—Short Form 10a	Children: at diagnosis and 6, 8, 11, 15 years end of paediatric care (< 18 years)	Self reported: from 8 years and older Parent reported: all ages
Adults		PROMIS Item Bank v1.0—Fatigue—Short Form 8a	18 years and then every 5 years	Self reported
Children	Social functioning	PedsQL—Questions social functioning and school functioning,	Children: at diagnosis and 3, 6, 8, 11, 15 years end of paediatric care (< 18 years)	Patient/parent reported
Children		PROMIS Pediatric Item Bank v2.0—Peer Relationships & PROMIS Parent Proxy Item Bank v.2.0—Peer Relationships	Children: at diagnosis and 6, 8, 11, 15 years end of paediatric care (< 18 years)	Self reported: from 8 years and older Parent reported: all ages
Adults		PROMIS Item Bank v2.0—Ability to Participate in Social Roles and Activities—Short Form 8a	Adults: 18 years and then every 5 years	Self reported
Adults		PROMIS v2.0 Brief Profile Sexual Function and Satisfaction (Female)	Adults: 18 years and then every 5 years	Self reported
Adults		PROMIS v2.0 Brief Profile Sexual Function and Satisfaction (Male)	Adults: 18 years and then every 5 years	Self reported
Children	Participation (education, work, leisure and sports)	PedsQL—school function (5 items)	Children: at diagnosis and 3, 6, 8, 11, 15 years end of paediatric care (< 18 years)	Patient/parent reported
Adults		PROMIS Item Bank v2.0—Satisfaction with Social Roles and Activities—Short Form 8a	Adults: 18 years and then every 5 years	Self reported
Adults		PROMIS Item Bank v2.0—Ability to Participate in Social Roles and Activities—Short Form 8a	Adults: 18 years and then every 5 years	Self reported

^a^Additional patient/parent reported tracking possible (“self service”)

### Selection of outcome measuring instruments

After four Delphi rounds the expert team reached consensus on the final set of measuring instruments shown in Table 3. For most domains, agreement was reached within the 1st and 2nd Delphi rounds. Some domains needed more discussion particularly those covered by patient reported outcomes measures (PROMs) covering multiple domains.

In these cases, the domains were discussed in combination because it was preferable to opt for one instrument that covered multiple domains with different subscales over different single domain instruments.

The PROMs that needed further discussion for children were Patient-Reported Outcomes Measurement Information System (PROMIS) Pediatric Instrument banks (Ped), the Pediatric Quality of Life Inventory (PedsQL) and the Pediatric Outcomes Data Collection Instrument (PODCI), which each cover several domains (pain interference, lower limb function, upper limb function, fatigue, emotional wellbeing, social functioning, self-care and participation) [15, 16]. Regarding Clinical Outcome Measures (COMs), the Functional Mobility Scale (FMS), the Gillette Functional Assessment Questionnaire (FAQ), 30 s walk test and the Medical Research Counsel (MRC) scales for manual muscle testing were discussed. The measuring instruments were again introduced in the 3rd and 4th Delphi round. Despite the PedsQL being conclusive for social functioning in the first Delphi round, the final Delphi round resulted in agreement on the use of the PROMIS Ped scales for all domains and consensus was reached for 28 measures (Table 3).

In contrast to the many options discussed for children with OI, the discussion in relation to adult care was more focused. Of the 19 pre-selected instruments, 8 were agreed on after the first Delphi round. With the second Delphi round, unanimous agreement was reached on 18 instruments. PROMIS was preferred over the Short Form (36) Health Survey (SF-36), due to the latter’s poor sensitivity in screening for the psychosocial issues and the time required resulting in a negative impact on the completion rates [17]. The possibility for computer adaptive testing (CAT) by PROMIS was seen as a significant advantage over SF-36. For the sake of using one instrument rather than two, PROMIS will also be used for the fatigue measurements. In a third Delphi round, consensus was reached on the final set of 24 outcome measures covering all domains (Table 3).

## Considerations per theme and domain

### Major events

#### Fractures

The expert team and focus groups expressed the need to address all aspects of bone fractures. Incidence, healing and type of treatment, as well as the mechanism of fracture (low impact vs high impact) in children will be reported. Incidence will be reported as the sum of clinically reported fractures, patient reported fractures and radiologically confirmed fractures, considering that not all fractures are always clearly visible on radiologic imaging. In daily practice, many patients are treated for clinical fractures without radiologic imaging or will manage minor fractures themselves without hospital visits and minimize the exposure to radiation.

#### Surgery

The focus groups defined surgeries as major life events in the majority of cases, as the severity of the disease and the quality of healthcare was determined by the complexity and frequency of surgery and the outcome. The expert team decided to record these events.

### Clinical status

#### Bone mineral density (BMD)

BMD, measured with Dual-Energy X-ray Absorptiometry scan (DXA-scan), is currently widely used as a substitute parameter for bone quality in OI and monitoring of medical treatment. Therefore a DXA-scan was selected as the preferred outcome measurement, despite its shortcomings of not taking into account altered body shape and the lack of a linear correlation to the fragility of the bones [18].

#### Spinal deformity

The expert team agreed to include the measurement of scoliosis and kyphosis with Cobb angles on total spine X-rays as spinal deformities are common in OI and severe malformations of the spine may lead to various other problems affecting quality of life.

#### Joints

The Beighton Score was selected to measure joint laxity during growth [19]. As laxity does not change in adulthood the Beighton Score will only be measured once at baseline.

#### Limb anomalies

Given the frequency of malalignment, the relation between bowing and fractures, the possibility for guided growth, and the need for surgery to improve function if significant malalignment is present, the expert team chose long standing axis X-rays to measure and report on limb alignment.

#### Short stature and growth

Physical appearance was considered an important issue during the focus group sessions, however no clinician or patient reported rating was found. Growth and stature by measuring height was determined to be the best way to express and monitor this domain.

### Function

#### Upper limb function

For the measurement of upper limb function and its impact on independence in daily life, the PROMIS Ped—upper extremity and PROMIS—upper extremity for adults were selected for children and adults [15]. Other PROMs and other COMs were felt to be too extensive for screening (e.g. ABILHAND-Kids, Bayley Scales of Infant Development, Peabody Developmental Motor Scales) or were not applicable to the majority of people with OI (e.g. the Brief Assessment of Motor Function (BAMF)).

#### Lower limb function

Measurement instruments from the literature search as well as those instruments suggested by the experts resulted in a choice of more than 30 instruments. There was consensus on using a combination of PROMs and COMs to describe clinical assessment as well as “real life” performance. Whilst feedback on the PROMIS Ped—mobility module to measure lower limb function was conclusive in the 2nd Delphi round, the choice of COM was not. The Gillette FAQ, BAMF, FMS, the timed up and go test and the 6 min, 1 min and 30 s walking tests were all discussed as possible options. The 30 s walking test was selected by the experts for both children and adults. It is the least burdensome, allows some measurement of progression and gives an outcome when walking is present [20]. For classifying functional mobility, the FMS was chosen for children, as it records the range of assistive devices a child may use and therefore provides information on the different assistive devices used in different environments [21].

For adults there was a good level of support among the experts for the PROMIS—physical functioning module as the PROM and the 30 s walking test as the COM.

#### Self-care

Age is a determining factor in this domain as adults have different goals in self-care compared to young children. For children the Functional Independence Measure for children (WeeFIM), PODCI, PedsQL, and PROMIS were discussed. As a relatively small percentage of children with OI have issues with self-care, (often due to upper extremity issues) the expert team concluded that screening for self-care problems in children could be addressed in the core set of measurements. Therefore, the expert team chose the PROMIS—upper limb module as screening instrument instead of the more detailed but time-consuming WeeFIM tool. If indicated, more specific instruments tailored to measure self-care skills are available.

In adults, the SF-36, PROMIS—upper extremity module and the Sunnaas index of ADL (SI)

were considered. The expert team felt that a more extensive self-care assessment was warranted for adults. As such, the SI was chosen over the SF-36 (with only one item on self-care) to complement the PROMIS—upper extremity module [15, 22].

### Quality of life

#### Pain

The focus groups reported pain as an important issue for individuals with OI as it affects daily life, mobility, participation, work life and social relationships. Pain was subdivided by the focus groups into acute pain such as in the case of fractures and chronic/persevering pain. Based on the strong support for PROMIS modules overall and no clear preference between PROMIS Ped—pain interference, PODCI and PedsQL, the expert team chose the PROMIS Ped—pain interference for children in the final outcome set. For pain intensity in children, the colored visual analog pain scale [23] was selected. In adults, both PROMIS—pain interference and pain intensity subscales were selected after the first 2 Delphi rounds.

#### Fatigue

The adult focus groups indicated fatigue was a notable problem, and it was also referenced in the child focus groups. For children, PROMIS Ped—fatigue as measurement tool was strongly preferred over PedsQL and PODCI.

For adults, the SF-36 vitality scale and PROMIS—fatigue remained after 2 Delphi rounds. Finally, the PROMIS—fatigue was chosen based on the strong support for the PROMIS modules overall [15].

#### Emotional well-being

Psychosocial issues are more prominent in OI compared to other disabilities [24]. Emotional well-being is a broad concept, which needed to be specified for the OI population. The expert team as well as the focus groups agreed on the importance of anxiety and mood. PROMIS Ped, PedsQL and PODCI contain some subscales covering emotional well-being. In the 2nd Delphi round there was a slight preference for using the PROMIS Ped scales and in the fourth Delphi round there was full agreement on using the PROMIS Ped—emotional distress anxiety and depression subscale to cover emotional well-being.

For adults the 3rd Delphi round resulted in strong support for the PROMIS—anxiety and depression subscales. The SF-36 (Emotional role functioning and mental health), the WHO QOL-BREF as well as the HADS were also subject to discussion but garnered low support in the first and second Delphi round.

#### Social functioning

Again, the PROMS PedsQL, PODCI and PROMIS Ped were suggested as the best options for the screening of social functioning in children with OI. Despite the PedsQL already being conclusive for social functioning in the first Delphi round, the final Delphi round resulted in agreement on the use of PROMIS Ped scales for all domains with PROMIS Ped -peer relationships replacing the PedsQL for social functioning.

For adults the SF-36, WHO Quality of Life—BREF (WHO QOL-BREF) social relationships, Female Sexual Functioning Index (FSFI), International Index of Erectile Function (IIEF), PROMIS—ability to participate, PROMIS—sexual function and satisfaction measures were all discussed. The PROMIS—ability to participate had strong support in the first Delphi round and the PROMIS—sexual function and satisfaction measures were added in the second Delphi round.

#### Participation

The focus groups as well as the expert group agreed that social functioning and participation are equally important and both items were retained. For children it was difficult to find an instrument for participation, which was not too time-consuming. While there was a preference to use the PROMIS Ped scales when possible, the school subscale of the PedsQL was optimal for participation and was selected [16]. Participation is also embedded in the PODCI but cannot be easily retrieved as a separate subscale.

For adults, participation is measured by the PROMIS—ability to participate in social roles and activities (already chosen to measure social functioning) as well as the PROMIS—satisfaction with social participation. Both had high support in the 2^nd^ Delphi round. The SF-36 -Mental Health domain—social function, was found to be less suitable in the 2^nd^ Delphi round.

## Discussion

The ICHOM methodology [14] was used to create a standardized set of outcomes based on the priorities of people with OI. An international group of health care providers, researchers and OI patient support organizations produced a consensus on a standard set for use in OI clinics around the world. All disciplines involved in care for OI participated as well adults and children with different types of OI were represented in order to measure what matters most to all people with OI. Individuals with OI are a very heterogeneous group. Creating a subset of outcome measures according to type/severity would be challenging. For example, a person categorized as type I may have a phenotype quite similar to someone identified as type IV. Creating subsets of outcome measures for each type or level of severity was considered. However, the goal of this project was to develop a minimal set of outcomes measures encompassing the most important domains for the majority of individuals with OI regardless of type worldwide. This standard set can be used to measure, analyze and improve outcomes achieved in the delivery of care. We recognize this set will require continual review.

There were differences between countries in terms of ranking the domains and this may be explained in part by cultural differences. However, the twelve domains rated most important were ranked as such quite consistently by the different countries and by both adults and child groups.

In order to structure the focus group meetings and their outcome, the items discussed were aggregated using the International Classification of Functioning, Disability and Health (ICF), a classification of health and health-related domains [13]. The items discussed were derived from a broad literature search. Since there was a wide range of outcome measures in the literature, efforts were made to create an overview of all reported outcomes. This was not a formal systematic literature review, however all items were discussed and supplemented if necessary by the expert group. Certain domains were not retained as the expert group observed these outcomes generally remained stable over time. Therefore, measuring them would not reflect an outcome that could be influenced. For example, dental problems, height in adults and mortality. These items are reported only once in the baseline characteristics of each patient.

The lead team made a pre-selection of the measuring instruments. These measuring instruments were available to the expert team for evaluation, in order to assess the face validity of the measuring instruments. It is possible that either the lead team or the expert team overlooked some relevant measuring instruments. For certain domains the actual content may have been slightly better covered using different measuring instruments, but in order to keep the minimal standard outcome set manageable we preferred measures that cover several subdomains over multiple different measuring instruments per domain. The expert team consisted of patient representatives, clinical and scientific experts in the field of OI. It did not contain lawmakers, hospital administrators or insurance companies since we did not want to focus on what could be possible but on what is necessary to measure in a minimal standard outcome set.

During this process of defining a standard outcome set for the care of people with OI, the guidelines for administering the outcome set has not yet been addressed. In the next phase of this project, clinical care teams from different countries worldwide will pilot the standard outcome set. The frequency and feasibility of administering the measurements will be carefully assessed when used in routine clinical management as well as in research in OI. Guidelines as to how to implement this standard set in both settings will be formulated and published once the results of the experiences of the pilot teams are analyzed.

## Conclusion

The international interdisciplinary Key4OI working group defined a standard set of recommended outcomes and associated measures that matter most to individuals with OI. This set of outcomes is recommended for use by interdisciplinary teams caring for people with OI. It is only by agreeing on a standard set of outcome measures for the comprehensive assessment of OI that comparison of outcomes across centers, needed for quality-improvement endeavors, comparative effectiveness research, and value-based healthcare reform can become a reality in the future.

## Data Availability

All data generated or analyzed during this study are included in this published article. The datasets used and/or analyzed during the current study are available from the corresponding author on reasonable request.
